# Alcohol-associated liver disease

**DOI:** 10.1172/JCI176345

**Published:** 2024-02-01

**Authors:** Bryan Mackowiak, Yaojie Fu, Luca Maccioni, Bin Gao

**Affiliations:** Laboratory of Liver Diseases, National Institute on Alcohol Abuse and Alcoholism, NIH, Bethesda, Maryland, USA.

## Abstract

Alcohol-associated liver disease (ALD) is a major cause of chronic liver disease worldwide, and comprises a spectrum of several different disorders, including simple steatosis, steatohepatitis, cirrhosis, and superimposed hepatocellular carcinoma. Although tremendous progress has been made in the field of ALD over the last 20 years, the pathogenesis of ALD remains obscure, and there are currently no FDA-approved drugs for the treatment of ALD. In this Review, we discuss new insights into the pathogenesis and therapeutic targets of ALD, utilizing the study of multiomics and other cutting-edge approaches. The potential translation of these studies into clinical practice and therapy is deliberated. We also discuss preclinical models of ALD, interplay of ALD and metabolic dysfunction, alcohol-associated liver cancer, the heterogeneity of ALD, and some potential translational research prospects for ALD.

## Introduction

**Figure 1:**
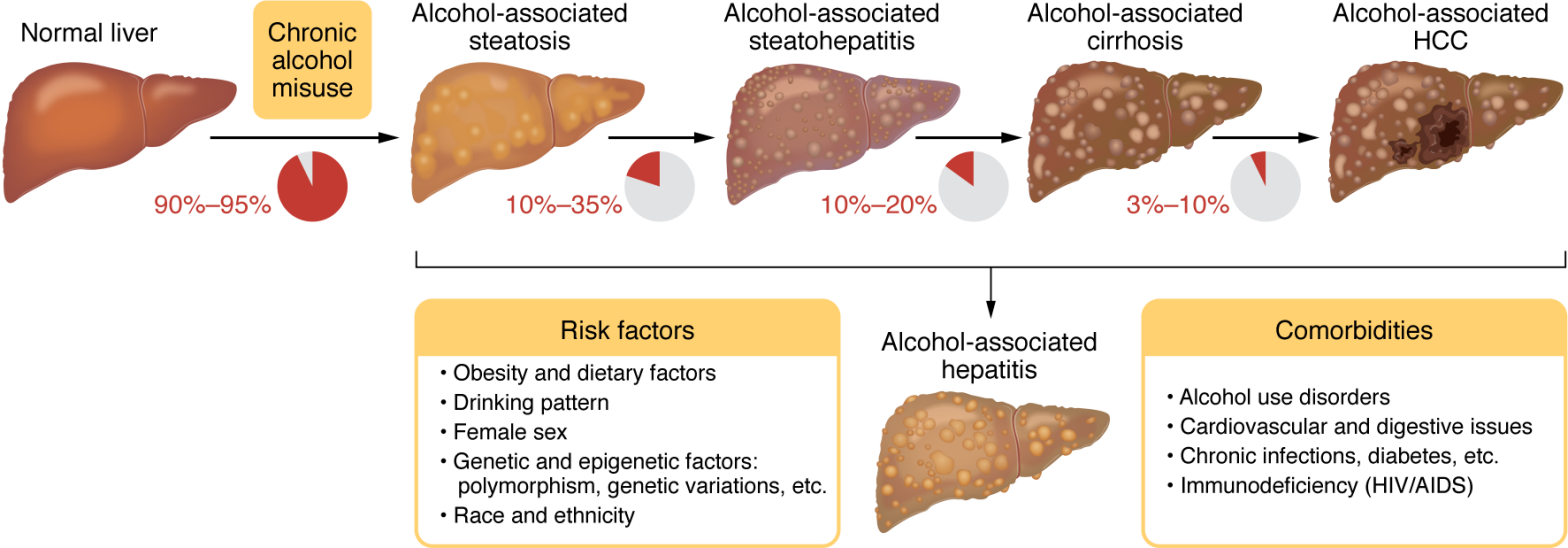
Spectrum of ALD, risk factors, and comorbidities. Almost all individuals who drink heavily (90%–95%) develop steatosis; some of them may develop more severe forms of ALD, including alcohol-associated steatohepatitis (ASH), cirrhosis, and hepatocellular carcinoma (HCC). Some patients with underlying ALD develop acute alcohol-associated hepatitis (AH) with the typical clinical syndrome jaundice. AH is often referred to as a severe form of AH that has a high short-term morality. ASH is diagnosed based on histology, while AH is diagnosed based on clinical symptoms. Many risk factors promote the development of the severe forms of ALD. Alcohol intake and comorbid factors synergistically promote the progression of ALD. Adapted with permission from *Gastroenterology* (4).

Alcohol-associated liver disease (ALD), which replaces the former name “alcoholic liver disease” to avoid the use of the stigmatizing word “alcoholic,” is one of most common chronic liver diseases worldwide, accounting for approximately 50% of cirrhosis in the USA (1–3). ALD includes an array of liver disorders, ranging from simple steatosis to more severe forms of pathological liver changes, including alcohol-associated steatohepatitis (ASH), cirrhosis, and hepatocellular carcinoma (HCC) (4) (Figure 1). In addition, patients with underlying ALD and excessive alcohol intake may develop acute alcohol-associated hepatitis (AH), an acute-on-chronic liver injury with prominent cholestasis that causes the typical clinical syndrome jaundice (5, 6).

**Figure 2:**
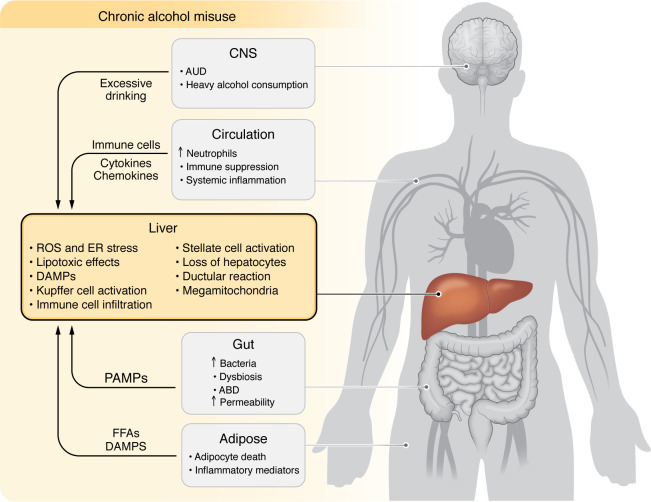
Pathogenesis of and interorgan crosstalk contribution to ALD. Excessive alcohol intake directly induces hepatocellular damage via multiple mechanisms. The crosstalk with several other organs, including brain-liver, gut-liver, and adipose-liver crosstalk, also contributes to ALD pathogenesis. Excessive alcohol consumption profoundly affects the immune system and immune cells, which also contributes to ALD progression. ABD, alcohol-associated bowel disease; AUD, alcohol use disorder; DAMP, damage-associated molecular pattern; PAMP, pathogen-associated molecular pattern; FFA, free fatty acid.

Alcohol is mainly absorbed in the small intestine and metabolized by the liver and other organs (7, 8), leading to disruption of liver metabolic homeostasis and forming the basis for ALD. Alcohol-associated steatotic liver, which replaces the former name “alcoholic fatty liver,” develops in more than 90% of individuals who are heavy drinkers and is characterized by fat accumulation in hepatocytes. Multiple mechanisms contribute to steatosis, including disruption of mitochondrial fatty acid β-oxidation, migration of lipids to the liver from extrahepatic organs, and alteration of lipid metabolism–associated transcription factors (4). ASH is a histologic diagnosis characterized by significant steatosis, inflammatory cell infiltration, chicken wire–like fibrosis, and hepatocyte ballooning, often with the formation of Mallory-Denk bodies. Patients with ASH progress to cirrhosis in 8%–20% of cases and patients with alcohol-associated cirrhosis progress to HCC in 3%–10% of cases. Diagnosis of AH is based on clinical presentation, including jaundice, right upper quadrant abdominal pain, fever, elevated serum bilirubin (>3 mg/dL), mildly elevated aspartate aminotransferase (AST) levels (>50 but <400 IU/L), and an AST/alanine aminotransferase (AST/ALT) ratio of >1.5 (5). In the clinic, the severe form of AH (sAH) has high short-term mortality and is typically referred to as AH, although moderate AH commonly exists (9, 10). There are still no FDA-approved drugs for sAH treatment, and clinical management of sAH involves treatment with oral corticosteroids (6). Importantly, patients who do not respond to corticosteroid treatment benefit from early liver transplantation (11). Most patients with mild-to-moderate ALD can recover after cessation of alcohol drinking (6). In addition, the most important determinant of survival in advanced ALD is whether the patient stops alcohol consumption, and, therefore, pharmacotherapy for alcohol use disorder (AUD) provides survival benefits in alcohol-associated cirrhosis (12–14). The pathogenesis and clinical management of ALD have been recently summarized in numerous reviews (4–6, 12, 15–18). In the current Review, we focus on several newly identified mechanisms (including organ crosstalk) that play key roles in ALD progression (Figure 2) and may lead to the discovery of therapeutic targets for sAH (Table 1). Recent advances in multiomics and other cutting-edge technologies have been actively applied in the field of ALD, especially sAH, which are briefly summarized. We also point out understudied areas in the ALD field, including alcohol-associated HCC (A-HCC), ALD heterogeneity, and differences in alcohol metabolism and ALD in individuals in Eastern and Western countries.

## Pathogenesis and therapeutic targets of ALD

### Hepatocyte death and regeneration.

Hepatocyte ballooning, degeneration, and acidophil bodies are the typic histologic features of hepatocyte injury in ASH, while severe ALD, including cirrhosis and sAH, exhibit significant loss of hepatocytes. Various mechanisms and factors have been implicated in induction of hepatocyte death in ALD, such as alcohol metabolism–associated endoplasmic reticulum stress, oxidative stress, proinflammatory cytokines (e.g., TNF-α), danger-associated molecular patterns, and dysregulation of autophagy, etc. (18). Several types of hepatocyte death have been reported and likely coexist in ALD, including apoptosis, necroptosis, pyroptosis, and ferroptosis (18). Hepatocyte death and impaired liver regeneration play an important role in promoting ALD progression and have been investigated as therapeutic targets. Selonsertib, a selective inhibitor of apoptosis signal-regulating kinase 1 (ASK1), has been tested for the treatment of patients with sAH, owing to its inhibition of hepatocyte apoptosis, but no beneficial effects were found (NCT02854631). Granulocyte colony-stimulating factor (G-CSF), which stimulates the bone marrow to produce granulocytes and hematopoietic stem cells, was thought to promote liver regeneration and was tested in clinical trials for sAH. However, the evidence for G-CSF stimulation of liver regeneration is insufficient (19), the clinical trial results for G-CSF were controversial in acute-on-chronic liver failure including AH, and G-CSF provided no survival benefit at 90 days in individuals with sAH, indicating that more evidence is required for further clinical investigation of G-CSF (20, 21). Finally, IL-22 may be an exceptional target that specifically protects against hepatocyte death and promotes hepatocyte proliferation without affecting immune cells owing to the restricted expression of IL-22 receptor on epithelial cells, including hepatocytes (22). The hepatoprotective effect of IL-22 has been demonstrated in a variety of liver injury models, including alcohol-induced liver injury (23, 24). A phase IIb clinical trial revealed that treatment of patients with sAH with recombinant IL-22 protein was well tolerated and had improved clinical parameters (25). Ongoing multicenter trials are being conducted to investigate IL-22Fc treatment for acute-on-chronic liver failure, including sAH (CTR20212657).

### Inflammation.

**Figure 3:**
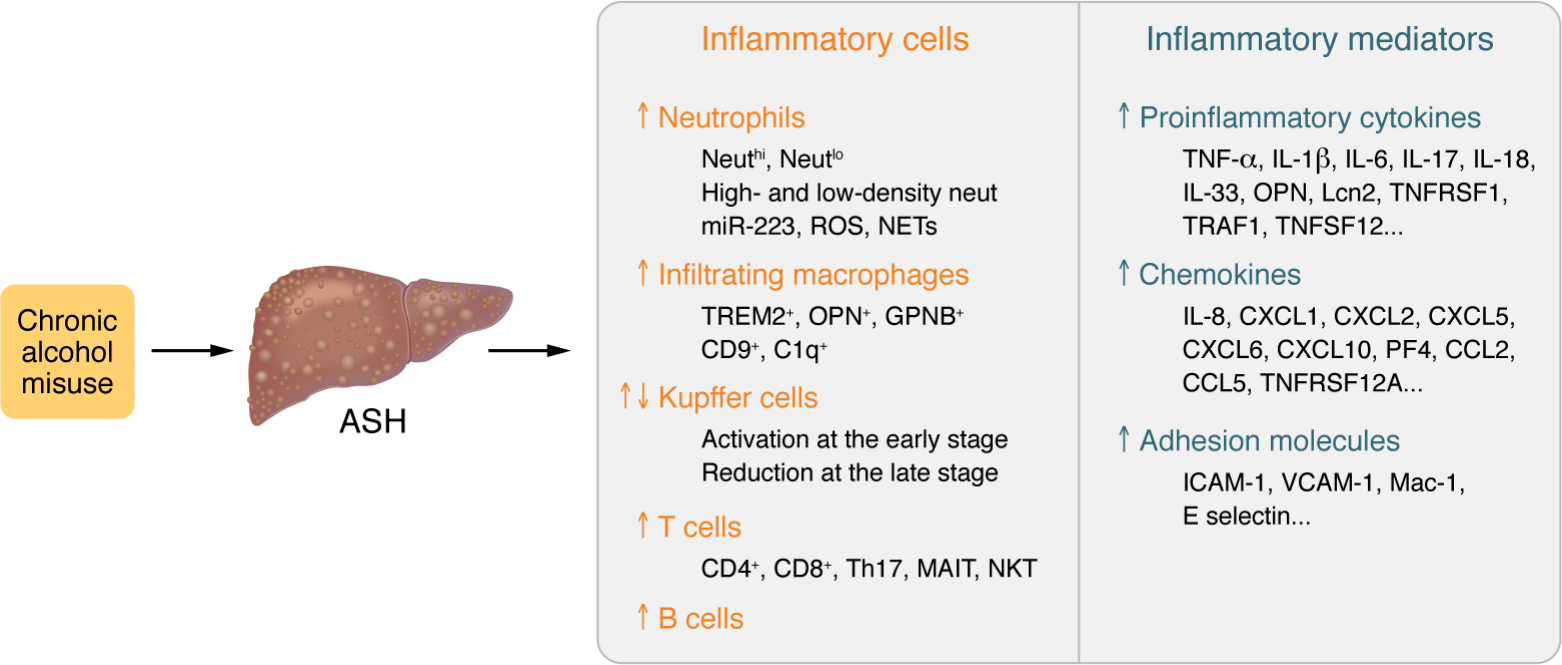
Inflammation in ALD. Alcohol-associated steatohepatitis (ASH) is characterized by hepatic infiltration of a large number of inflammatory cells, with predominant neutrophil and macrophage infiltration. Kupffer cells are activated at the early stage of ALD but are markedly reduced in the late stages of ALD, such as cirrhosis. ALD is also associated with infiltration of a significant number of T cells, but their subtypes and functions have not been well characterized. ALD, especially severe AH, is associated with infiltration of B cells and massive antibody deposition. The subsets and functions of inflammatory cells will be likely identified by single-cell and spatial transcriptomics and multiplex immunofluorescent staining analysis over the coming years. ASH is also associated with elevation of a large number of proinflammatory cytokines, chemokines, and adhesion molecules, which have overlapping functions and synergistically promote liver inflammation.

Inflammation acts as a key factor driving ALD progression to steatohepatitis, cirrhosis, and HCC (17); many different cell types and inflammatory mediators participate in the inflammation underlying ALD (Figure 3). Paradoxically, alcohol is a well-known immunoregulator that strongly inhibits the immune system, causing the increased host susceptibility to bacterial and viral infections (26). The major factors that trigger ALD inflammation include hepatocyte death, increased gut permeability, and disrupted intestinal bacterial homeostasis (dysbiosis) (17). ALD inflammation is characterized by infiltration of neutrophils and macrophages, as well as activation of Kupffer cells and other types of immune cells (17), which play a dominant role in the pathogenesis of ALD. The detrimental effects of macrophages in ALD are likely due to production of a variety of inflammatory mediators (17), while neutrophils exacerbate ALD by producing ROS, inflammatory mediators, and neutrophil extracellular traps (27, 28). On the other hand, macrophages and neutrophils play some beneficial roles in ameliorating ALD by promoting liver regeneration, fibrosis resolution, and antibacterial immunity, etc. (17, 18). Significant infiltration of T cells is also observed in ALD, especially in the alcohol-associated cirrhosis, but their exact roles have not been well characterized in patients with ALD (17). Emerging evidence suggests that T cells have important profibrotic roles in metabolic dysfunction–associated steatotic liver disease (MASLD) (29), so it will be important to examine whether T cells also contribute to liver fibrogenesis in ALD. Interestingly, a negative correlation of intrahepatic neutrophils with intrahepatic CD8^+^ T cells was observed in patients with sAH, and two distinct histopathological phenotypes were defined based on liver immune phenotyping, suggesting a separate mechanism driving liver injury and/or failure in these patients (30). A significant number of B cells are also seen in sAH, which is accompanied by massive antibody deposition and evidence for complement activation in hepatocytes, all of which play an important role in promoting liver injury in sAH (31). In addition, many other types of cells may also play a role in modulating ALD disease progression in preclinical models, including NKT cells, Th17 cells, and mucosal-associated invariant T cells, but their functions in ALD pathogenesis are not clear. Moreover, many proinflammatory mediators are upregulated and likely synergistically promote disease progression in ALD (17, 18).

Given its important role in the pathogenesis of ALD, inflammation has been actively investigated as a therapeutic target for sAH therapy. Steroids have been used to treat sAH since 1970s, and emerging data suggest that steroid treatment improves short-term survival in some patients with sAH without affecting long-term survival (32). Inhibition of specific inflammatory targets (e.g., TNF-α, IL-1) has been investigated for sAH therapy, but this approach did not achieve good clinical benefit (33–35), which is likely because sAH is associated with elevation of many inflammatory mediators that have overlapping functions (Figure 3) (17). The next question is whether we can directly target inflammatory cells to treat sAH. Significant numbers of infiltrating macrophages are detected in sAH, and these cells likely drive sAH inflammation and are potential targets for sAH therapy (17). Inhibition of macrophage infiltration by cenicriviroc, an oral dual chemokine receptor CCR2/CCR5 antagonist, generated some beneficial effects in preclinical models of MASLD (36) and ALD (37). However, recent studies have identified many subsets of macrophages, with some of them playing an important role in promoting liver repair and fibrosis resolution (38, 39), thus, selective inhibition of inflammatory macrophage infiltration may achieve better clinical outcomes for ALD treatment. Interestingly, binge alcohol intake or recent excessive drinking elevated circulating neutrophils and subsequently increased hepatic neutrophil infiltration and liver injury, which can be inhibited by blockade of C-X-C motif chemokine receptors 1 and 2 (CXCR1 and CXCR2) in preclinical models (27, 28, 40–43). Targeting neutrophils for the treatment of sAH has not been explored clinically, but inhibition of CXCR1 and CXCR2 and other therapies that modulate neutrophils deserve further investigation. In addition, several other types of immune cells (e.g., T cells, NKT cells, mucosal-associated invariant T cells) have been implicated in the pathogenesis of AH (17); however, more clinical studies are required to clarify their functions in AH and evaluate their potential as therapeutic targets for AH therapy.

### Gut dysfunction and dysbiosis.

Alcohol misuse can cause a profound impairment of intestinal functions, including a disease called alcohol-associated bowel disease (44). Alcohol-induced intestinal dysfunctions include malabsorption of nutrients, reduced villus-to-crypt ratio restricted to the duodenum, increased intestinal permeability, reduced production of antimicrobial molecules, increased mucus thickness, a striking diminution of mucosal immune cells, and gut microbiome-related changes (44). In general, reduction of immune cells in the intestine is a unique feature of alcohol-associated bowel disease, which is different from other intestinal diseases (e.g., celiac disease, inflammatory bowel disease) characterized by intestinal inflammation (45). Alcohol-mediated reduction of intestinal immune cells results in intestinal immune dysfunction and subsequently contributes to gut barrier disruption (44). However, how chronic alcohol consumption exactly affects different pro- and antiinflammatory immune cell populations in different intestinal tracts still remains unclear. Moreover, alcohol misuse and ALD are associated with small intestinal bacterial overgrowth, alterations of gut microbiota (“dysbiosis”), and bacterial translocation (46, 47). Gut dysbiosis was first reported in rats (48) and later in mice (49) after chronic ethanol exposure. In mice, chronic ethanol feeding increased the abundance of *Bacteroidetes* and *Verrucomicrobia* bacteria but decreased the abundance of lower *Firmicutes*, and these changes were associated with downregulation of antimicrobial *Reg3g* and *Reg3b* gene expression in the proximal small intestine (49). The Schnabl group later found that cytolysin secreted by *Enterococcus faecalis* causes hepatocyte death and liver injury (50). Increased fecal numbers of *E*. *faecalis* were found in patients with sAH, and the presence of cytolysin-positive (cytolytic) *E*. *faecalis* correlated with the severity and mortality of sAH (50). Colonization of gut microbiome from the feces of patients with sAH induced liver injury in mice, which can be ameliorated by bacteriophages that specifically target cytolytic *E*. *faecalis* (50). Emerging evidence suggests that ALD-associated changes in intestinal fungi also contribute to the pathogenesis of ALD by producing toxins and metabolites (51) and that the intestinal virome is altered in AH (52), but further study is required in these areas.

Restoring intestinal epithelial integrity and antimicrobial function and correcting dysbiosis are attractive strategies for ALD. Zinc is critical for maintenance of intestinal barrier function (53), and zinc deficiency is associated with ALD (54) and exacerbates ALD in preclinical models (55). Zinc supplementation has been included in the anti–IL-1 trial for sAH, but this trial did not improve sAH (34). In addition to protecting against liver injury, IL-22 also protects against gut epithelial injury, promotes gut epithelial cell regeneration, and restores intestinal immunity (56). Activation of IL-22 in the gut via bacteria engineered to produce IL-22 or produce aryl-hydrocarbon receptor agonists that upregulate IL-22 protects against ALD in mice (57, 58). Moreover, activation of intestinal epithelial aryl hydrocarbon receptor by microbial tryptophan metabolites improves alcohol-mediated gut barrier dysfunction and has potential as a therapeutic target for ALD (59, 60). Targeting microbiome and mycobiome toxins have been actively investigated for the treatment of ALD (50, 51). Fecal microbiota transplantation as well as antibiotics, probiotics, and prebiotics have been tested or proposed as gut microbiome-centered therapies for ALD, but the results are inconsistent (61–64). Given the high heterogeneity of the gut microbiome in humans, the descriptive nature of the microbiome studies so far, and the lack of a definition for a “healthy” microbiome (65), it is difficult to have conclusive results. It is unlikely that targeting the gut microbiome alone will be sufficient to treat ALD in all patients.

### Ductular reaction.

Ductular reaction (DR) is associated with advanced ALD and is characterized by an increased number of cholangiocytes along with inflammatory cell infiltration and loss of hepatocytes (66). The origin of the expanded cholangiocytes is controversial, and multiple origins have been proposed, including cholangiocyte proliferation, hepatic progenitor cell differentiation into cholangiocytes, and dedifferentiation of hepatocytes toward a cholangiocyte-like phenotype (66). Thus, targeting DR to preferentially differentiate hepatic progenitor cells into hepatocytes is a potential strategy for the treatment of advanced ALD. Several drivers of DR have been identified, including modulation of biliary NF-κB activity, long noncoding RNA *ACTA2-AS1*, mTOR activation, CXCR4-mediated hepatocyte dedifferentiation, and neutrophil or macrophage infiltration (67–76). Of these, inflammation-mediated DR, mTOR activation, and biliary NF-κB activity alterations seem to be present in human ALD samples, indicating their translational significance. However, targeting the mTOR and NF-κB pathways specifically in cholangiocytes is difficult and, therefore, inhibiting inflammatory exacerbation of DR may hold the most potential as a therapeutic approach. Regardless, DR is associated with worse prognosis in ALD, and therapeutics that reverse DR, hepatocyte dedifferentiation, and the cholestatic phenotype hold potential for ALD treatment.

### Hepatic mitochondrial dysfunction.

Hepatocytes are rich in mitochondria, which play important roles in glucose, lipid, and protein metabolism as well as ROS homeostasis. A wide range of studies have found that heavy alcohol consumption causes impairment of mitochondrial biogenesis, mitochondrial DNA damage, and subsequent oxidative stress and cell death (77, 78). In addition, formation of megamitochondria in hepatocytes has been a known effect of heavy alcohol use since the 1970s (79), but how these changes are related to ALD progression were unknown until recently. The Ding lab demonstrated that alcohol consumption decreased hepatic dynamin-related protein 1 (DRP1), a protein involved in mitochondrial fission, and induced megamitochondria in cells and a mouse model of ALD (80). Patients with sAH have decreased hepatic DRP1 that is associated with increased accumulation of megamitochondria in the liver, and genetic deletion of the *Drp1* gene markedly exacerbates ALD in mice, supporting the role of mitochondrial dysfunction in ALD progression (80). Additionally, another study suggests that activation of hepatic activating transcription factor 4 acts as a driver of alcohol-impaired mitochondrial biogenesis and respiratory function (81). Defective mitochondrial respiratory function can provoke elevated ROS production and subsequently sensitize hepatocytes to death, a key event in ALD progression (79). Collectively, these recent studies suggest that modulating mitochondrial homeostasis in ALD is a potential therapeutic strategy and requires further characterization.

### Other potential mechanisms and therapeutic targets.

Over the last 20 years, many molecular mechanisms have been identified that may contribute to the pathogenesis of ALD (18), but translation of these mechanisms to therapeutic targets needs further attention. For example, alcohol consumption causes adipose inflammation, lipolysis, and damage, which likely contribute to ALD pathogenesis (82, 83). Moreover, dysregulation of lipid metabolism contributes to MASLD progression by inducing hepatocyte death and has been actively investigated as a therapeutic target for MASLD (84), but its role in sAH and whether it can be used as a therapeutic target for ALD is unclear. An endogenous cholesterol derivative, 25-hydroxycholesterol 3-sulfate (larsucosterol), was found to inhibit liver lipid accumulation and improve cell survival by inhibiting DNA methyltransferases, which has shown promise in a phase IIa clinical study for moderate and sAH (85). Several therapies targeting ROS have shown mixed effects for sAH treatment, with N-acetyl cystine (NAC) providing no benefit (86, 87) and metadoxine providing modest survival benefits (88). Autophagy has been shown to play an important but complex role in the pathogenesis of liver diseases, including ALD (89, 90), but has not been tested clinically as a therapeutic target for ALD due to its highly complex, cell-specific roles (91). Many miRNAs have been found to modulate ALD disease progression, but the application of these miRNAs as therapeutic targets in ALD treatment is still at the early stage of investigation (92). Further translational studies are required to test the therapeutic potential of autophagy modulators and miRNAs for ALD treatment.

## Application of cutting-edge technologies in ALD

### Multiomics analysis of ALD.

The emergence and application of -omics (genomics, transcriptomics, proteomics, metabolomics) technologies to ALD in preclinical and clinical models over the past 20 years have provided a wealth of data about the genetic polymorphisms and signaling pathways that drive the progression of ALD (Table 2 and Table 3). Human GWAS identified risk factors for ALD, including polymorphisms in *PNPLA3*, *MBOAT7*, *TM6SF2*, *MARC1*, *HNRNPUL1*, *HSD17B13*, and other genes (Table 4), many of which are also risk factors for other types of liver diseases (further detailed in *Heterogeneity of ALD* below) (93–99).

One of the first microarray studies in ethanol-fed mice identified numerous pathways altered by ethanol, including fatty acid metabolism, glutathione metabolism, and cytokine signaling, providing several previously unknown ethanol-regulated genes for further study (100). In addition, the application of transcriptomics to human ALD samples has provided much more information about the pathogenesis of ALD, mostly due to the inability of the current preclinical ethanol-feeding models to recapitulate all hallmarks of human ALD. Early transcriptomic studies of human AH identified dysregulation of the TNF receptor superfamily member 12 A (TNRSF12A), and integration of this data set with microarray analysis of the chronic-plus-binge mouse model identified fat-specific protein 27/cell death inducing DFFA-like effector C (FSP27/CIDEC) as a driver gene of ASH in mice and humans (101, 102). Several other mouse transcriptomic studies have enhanced our understanding of ALD, including the role of gasdermin D–mediated pyroptosis and neutrophil cytosolic factor 1–mediated oxidative stress, among others (30, 103–107). Expanding access to RNA-Seq technologies has led to more transcriptomic studies in mouse and human ALD, especially over the past 5 years. One of the key studies by Argemi et al. integrated GWAS and methylomic data with RNA-Seq data to identify hepatocyte nuclear factor 4α (HNF4α) dysregulation and subsequent hepatocyte dedifferentiation as a major contributor to ALD progression (108).

Integration of transcriptomics with other -omics technologies, including proteomics and metabolomics, has led to big leaps in our understanding of ALD and the identification of potential biomarkers. By applying proteome microarrays, one recent study found that livers from patients with sAH contain a large number of autoantibodies that are not present in circulation, and deposition of these antibodies likely participates in sAH inflammation (31). Studies combining transcriptomics with proteomics have identified and validated potential plasma biomarkers of ALD (109, 110). Several other studies investigating circulating biomarkers for ALD have identified lncRNAs, small RNAs, metabolites, proteins, and the circulating transcriptome as potential identifiers of ALD (111–118). Further validation of these potential biomarkers and integration with ALD severity scoring will be essential for proper diagnoses and potentially could be expanded to determine whether patients will respond to a specific therapy once more ALD therapeutics become available. One study used liver biopsy transcriptomes to determine whether patients would respond to corticosteroid therapy, and application of circulating biomarkers to the potential for response to treatment will begin to lead to personalized medicine in the future (119).

The advances from simple RNA-Seq toward single-cell and spatial transcriptomics will likely drive the next wave of discoveries surrounding ALD. Indeed, by utilizing scRNA-Seq analysis, two studies have identified new cell subpopulations that drive fibrosis and inflammation in the mouse AH model and human alcohol-associated cirrhosis (120, 121). In addition, a previous study using microdissection to determine gene expression in different spatial areas found that increased yes-associated protein (YAP) signaling in hepatocytes leads to biliary transdifferentiation as a mechanism of AH (122). Epigenetics in ALD has also been received much attention in the past (123, 124), while gene splicing is another area that has been investigated recently. For example, Hyun et al. recently found that epithelial splicing regulatory protein 2 plays an important role in controlling hepatocyte reprogramming in AH (125). Another study reported that serine-arginine-rich protein kinase 2, a key kinase controlling alternative splicing, is activated in hepatocytes in response to alcohol and promotes ALD in mice (126).

### Multiplex immunofluorescence staining analysis of ALD.

Advances in protocols for staining and visualization of formalin-fixed paraffin-embedded (FPPE) tissues have enabled labs with little to no specialized equipment to conduct multiplex immunofluorescence staining. This allows users to investigate the spatial distribution of cell types and proteins of interest through staining and visualizing more than 12 different proteins (127). This technique has been widely applied to investigate the tumor microenvironment and other types of liver disease but has only recently been utilized to interrogate the pathology of ALD (30, 31, 71). By using multiplex immunofluorescence staining, we detected high numbers of macrophages near DR in sAH, suggesting that macrophages play a role in promoting DR (127). Indeed, a study from an experimental model revealed that macrophages promote ductular cell repair and proliferation after acute bile duct injury (128).

## Preclinical models of ALD

Chronic alcohol feeding via either voluntary intake or intragastric tube has been used to induce ALD in animals over the last 4 decades (129–132) (Table 5). Such chronic feeding induces steatosis and liver injury with activation of macrophages but lacks neutrophil infiltration (a hallmark of AH). In 2010, we introduced binge ethanol intake into the chronically ethanol-fed mice, which causes significant neutrophil infiltration and liver injury (23, 133). Thereafter, this chronic-plus-binge model has been widely used in the field and is considered as a model for mild AH over the last decade (134, 135). In addition, many “second-hit” models of liver injury in combination of ethanol with other insults have also been developed (136). By using these models together with analysis of human ALD samples, many novel molecular pathways and mechanisms involved in pathogenesis of ALD have been identified (134, 135). Recently, the combination of alcohol and Western diets has been actively tested in mouse models. Single or multiple binges of ethanol exacerbate liver injury in mice fed a high-fat diet (HFD) (102, 137, 138) or Western diet (139). Chronic alcohol feeding via intragastric tube has also been applied in mice fed a Western diet (140), but voluntary ethanol feeding in combination with HFD is challenging due to decreased food intake in ethanol groups compared with the pair-fed control groups (our unpublished data). Interestingly recent studies reported that ethanol in drinking water and/or binge ethanol intake exacerbated liver injury in mice fed the Western diet (141, 142), which is required further characterization.

## Interplay of ALD and metabolic dysfunction

There have been recent changes in the nomenclature for liver disease, with steatotic liver disease, which encompasses all types of fatty liver diseases, now termed MASLD, which replaces the stigmatizing “nonalcoholic fatty liver disease” (NAFLD), and a new category, called MetALD, which includes patients with MASLD who also have significant alcohol consumption (143, 144). The combination of metabolic dysfunction and heavy alcohol consumption in this unique patient population exhibits overlapping and distinct mechanisms of liver disease progression which have been recently reviewed (145). As the rates of metabolic dysfunction are increasing in populations across the world, MetALD should be a priority for study going forward.

## Alcohol-associated cirrhosis

Alcohol-associated cirrhosis causes an estimated 25% of global deaths due to liver cirrhosis and up to 40% in certain areas of Europe (146). In addition, deaths due to alcohol-associated cirrhosis in the United States have risen in younger individuals and are projected to rise precipitously up to 2040 (1, 147). While alcohol-associated cirrhosis represents a distinct etiology of cirrhosis, its diagnosis and treatment are relatively similar to MASLD- and viral-initiated cirrhosis, which have been recently reviewed (148). It is essential to treat the underlying AUD in patients with alcohol-associated cirrhosis to improve outcomes (14).

## Alcohol-associated liver cancer

Recent epidemiological data revealed that alcohol contributes to an estimated 19% of liver cancer deaths globally, and the age-standardized death rate of alcohol-associated liver cancer increased by 0.53% annually in the past few years (149, 150). Although alcohol drinking is a well-known risk factor for liver cancer, especially HCC, A-HCC is poorly characterized compared with HCC caused by other etiologies (151). Among patients with ALD, the annual incidence of A-HCC is 5.6 cases per 1,000 person-years (152). Moreover, patients with A-HCC tend to be diagnosed with advanced stage disease compared with patients with other etiologies of HCC (153, 154), which is partially due to a lack of access to early screening in populations with ALD (155). Of note, compared with the general population, the relative risk (RR) of HCC was 2.4 for AUD alone, and the presence of cirrhosis increased the RR of developing HCC among people with AUD to 22.4 (156). Cirrhosis is a necessary intermediate step for A-HCC development and amplifies the overall risk for carcinogenesis in patients with ALD (157, 158). Analysis of causes of death for Danish patients with ALD revealed that the majority of deaths are due to the liver disease itself in the 5 years after diagnosis, after which extrahepatic cardiovascular, cancer, AUD-related deaths become more common, while individual cancers, including A-HCC, are minor contributors to ALD-related mortality (159). However, improved ALD treatments in the future will likely increase longevity of patients with ALD, which may lead to an increase in a number of patients with A-HCC.

Currently, there are no ideal animal models for A-HCC study. Despite most available A-HCC mouse models combining the carcinogenic agent N-nitrosodiethylamine with long-term ethanol feeding (160), their clinical relevance to human A-HCC is still questionable. Developing appropriate preclinical models of A-HCC might improve translation of basic science into clinical practice, which may provide a better understanding of hepatocarcinogenesis in patients with ALD.

## Heterogeneity of ALD

ALD is a heterozygous disease characterized by a spectrum of disorders, and this heterogeneity likely contributes to the failing of various clinical trials for ALD treatment (4). A better understanding of how factors such as genetics, drinking pattern, dietary effects, bacterial infection, and comorbidities alter mechanisms behind the development and pathogenesis of ALD is essential and may lead to personalized treatments for ALD (4).

### Genetic heterogeneity in ALD.

As described above, GWAS have been utilized to link genetic associations with risks of developing ALD and subsequent outcomes (Tables 3 and 4) (the full gene names are listed in the Table 4). The two most robustly replicated single nucleotide polymorphisms, rs738409(C>G) in *PNPLA3* and rs58542926(C>T) in *TM6SF2*, are closely related to the increased risk for developing the entire spectrum of ALD (161). As lipid turnover–related genes, *PNPLA3* rs738409 variant and *TM6SF2* rs58542926 variant are involved in abnormal hydrolysis of triglycerides and very-low-density lipoprotein secretion (162), which are significantly associated with a higher risk for alcohol-associated cirrhosis and may predispose patients with cirrhosis to A-HCC (93, 163). *MBOAT7* rs641738 and *NCAN* rs2228603, two common variants related to the development and severity of MASLD as well as liver fat content (164, 165), are found to increase the risk for cirrhosis and HCC development in ALD (93, 166). Many of the genes associated with increased risks of ALD progression (Table 4) are linked to increases in liver fat content, but exactly how these changes lead to carcinogenesis in the setting of cirrhosis requires further study. Additionally, *WNT3A-WNT9A* rs708113 was recently identified as a susceptibility locus for A-HCC; however, more evidence is needed to clarify its gene-alcohol interactions (161). Moreover, polymorphisms of ethanol metabolic genes (e.g., ADH1C*1 and ALDH2*2) may also influence an individual’s susceptibility to A-HCC (167–169). Conversely, some newly identified genetic polymorphisms may play protective roles in ALD progression. The rs2242652 germline variant in *TERT* and variants of several lipid metabolism–related genes, including *MARC1* rs2642438, *APOE* rs429358, *HSD17B13* rs72613567, and *LPL* rs13702, are associated with reduced risk for cirrhosis or HCC development in patients with ALD (94, 170–172). There are a few things to keep in mind with these studies. The first is that some of these genetic association studies have been conducted in relatively small populations, and replication of these findings in larger cohorts will be important. In addition, the levels of evidence for the association of each of these polymorphisms with ALD vary across the different disease stages. Finally, many of the genetic risk factors for ALD are different from those for AUD, with the finding that only a subset of patients with AUD ever progress past steatosis to more severe stages of ALD (173).

### Comparing alcohol metabolism and ALD in Eastern and Western populations.

More than 90% of ingested alcohol is metabolized into acetaldehyde by oxidative enzymes alcohol dehydrogenase (ADH) and to much lesser extent by cytochrome P450 2E1 and catalase. Acetaldehyde is converted further into acetate by mitochondrial aldehyde dehydrogenase 2 (ALDH2). The traditional notion of the liver as a major site for ethanol metabolism is challenged by our recent study showing that deletion of the liver *Aldh2* gene reduced blood acetaldehyde clearance only by approximately 30% compared with that in global *Aldh2*-knockout mice (7), suggesting many other organs that express ALDH2 also contribute to acetaldehyde metabolism. Additionally, alcohol can also be metabolized by a nonoxidative pathway to generate lipophilic fatty acid ethyl esters (FAEEs), which also promotes liver injury (174); however, more studies are required to clarify the role of FAEEs in ALD pathogenesis.

People from Asia and Western countries have significant differences in ethanol metabolism due to the polymorphisms in *ADH* and *ALDH2*. For example, 30%–40% of individuals from eastern Asia have inactive *ALDH2* polymorphisms (ALDH2*2/1, ALDH2*2/2) (175), and approximately 70% of these individuals have *ADH* polymorphisms with higher ADH activity (176). Thus, many Asians drink less alcohol but generate much higher levels of acetaldehyde, exhibit flushing, and generate lower levels of ethanol-derived acetate compared with the people from Western countries, which may differently affect ALD development and progression (177). Mounting evidence suggests that ethanol consumption among those harboring the ALDH2*2 polymorphism is rising, and identifying any differences in ALD pathogenesis in this population will become increasingly important (178, 179). Preclinical models revealed that mice with ALDH2 deficiency have more inflammation and fibrosis and greater immunosuppression but lower levels of steatosis and serum ALT compared with control mice after ethanol intake (174, 180, 181). Therefore, the pathogenesis of ALD between the East and the West may exhibit some differences, and we may need different diagnosis guidelines and therapy for those with inactive ALDH2 and/or greater ADH activity in Asia.

### Sex disparities in ALD.

There is a clear sex disparity regarding the epidemiology of ALD. Women are more susceptible to ALD than men with the same amount of alcohol intake, although the exact mechanisms are still unclear (151). The RR of developing ALD is 3.7 in men and 7.3 in women (182). The risk of alcohol-related cirrhosis in male and female heavy drinkers (at age 40 years, with 10 drinks/day for more than 15 years) is 3.1% and 4.7%, respectively (183, 184). Additionally, compared with men, women with AH are generally younger but have higher rates of AH-related complications, comorbidities, and mortality (185). Moreover, even though more men overall have ALD, the increase in ALD mortality is more rapid among women than men (186–188). Therefore, identifying gender-related mechanisms underlying the higher risks of ALD in women should be a priority for further study.

## Conclusions and translational research prospects

Despite of extensive research on ALD over the last four decades, there are still no FDA-approved drugs for ALD. We have detailed some potential translational research prospects for ALD in Table 6, but we believe that new cutting-edge technologies applied to samples from patients with ALD and experimental models in many other areas will also yield valuable information for ALD pathogenesis and treatment. Hepatocyte death, impaired liver regeneration, inflammation, DR, and organ-liver crosstalk all play key roles in promoting ALD and represent areas for therapeutic development. A better understanding of the gut-liver axis during ALD progression is needed, and future studies should rigorously investigate intestinal immunity–microbiome interactions in the context of alcohol use. In addition, all current mouse models of ALD generate mild-to-moderate liver injury, inflammation, and fibrosis or fibrotic responses. Even combination treatment of alcohol with other insults did not recapitulate the full spectrum of human ALD in mice. Many factors may contribute to the resistance of severe ALD in mice, including but not limited to much faster ethanol metabolism, low neutrophil count, and lack of the key neutrophil chemokine CXCL8 (IL-8) and CXCL6 in mice (189). Mice with genetic modification of these factors should be tested for ALD. In addition, combination of alcohol with different diets in preclinical models should be tested, which may identify dietary factors that play an important role in ALD. In addition, the recent advances in rapid in vivo multiplexed editing of the adult mouse liver using CRISPR/caspase-9 will likely help to identify how different systems interact in ALD in preclinical models (190). Finally, development of ALD biomarkers is also essential for the early diagnosis of clinically “silent” ALD, allowing early intervention with AUD therapy to decrease alcohol consumption and potentially reverse ALD in some patients.

## Author contributions

BM and YF wrote the manuscript and contributed equally. LM wrote the gut dysfunction and dysbiosis parts of the manuscript and edited it. BG wrote and edited the manuscript and supervised the whole project.

## Figures and Tables

**Table 6:**
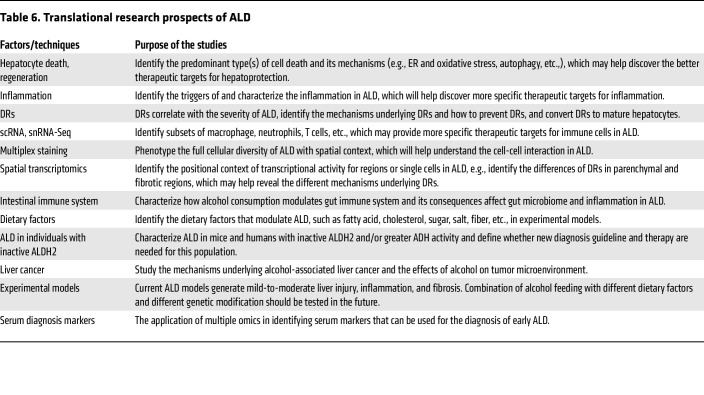
Translational research prospects of ALD

**Table 5:**
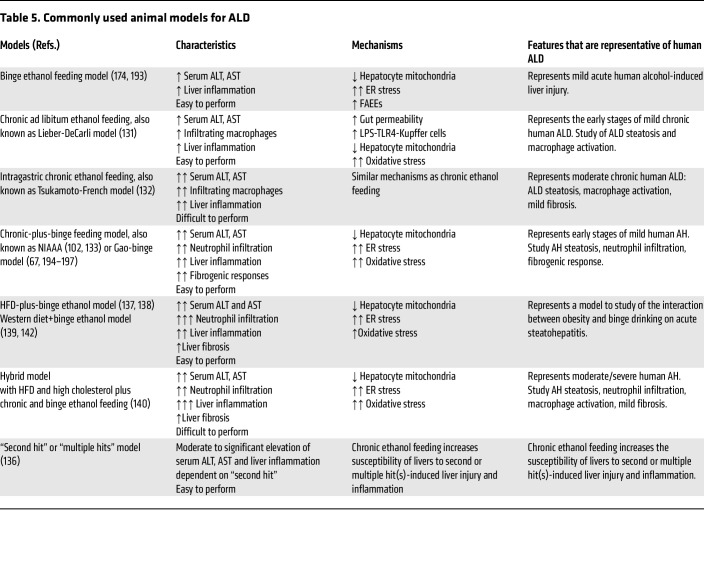
Commonly used animal models for ALD

**Table 4:**
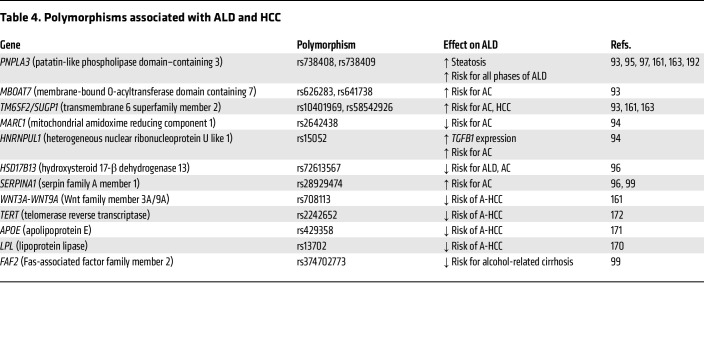
Polymorphisms associated with ALD and HCC

**Table 3:**
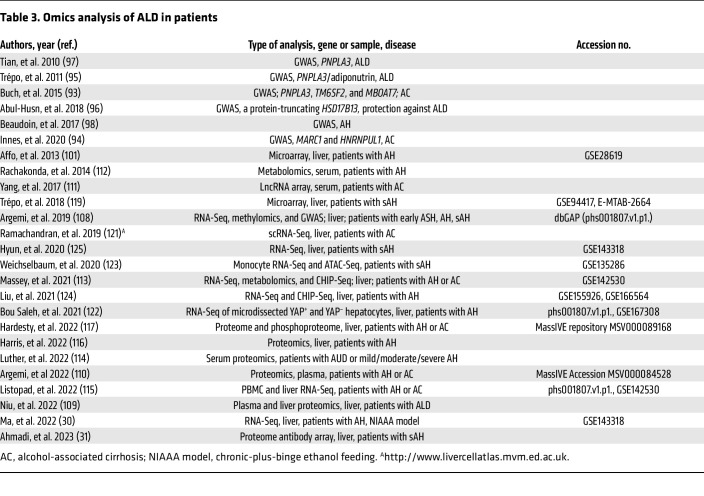
Omics analysis of ALD in patients

**Table 2:**
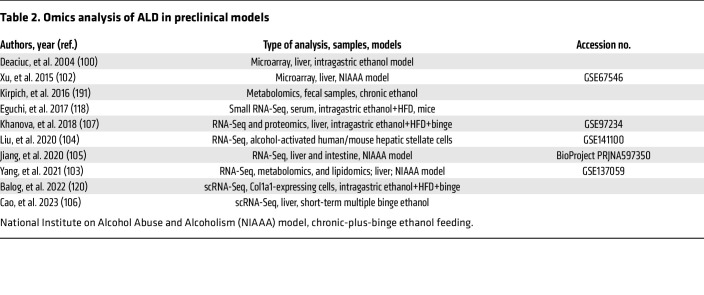
Omics analysis of ALD in preclinical models

**Table 1:**
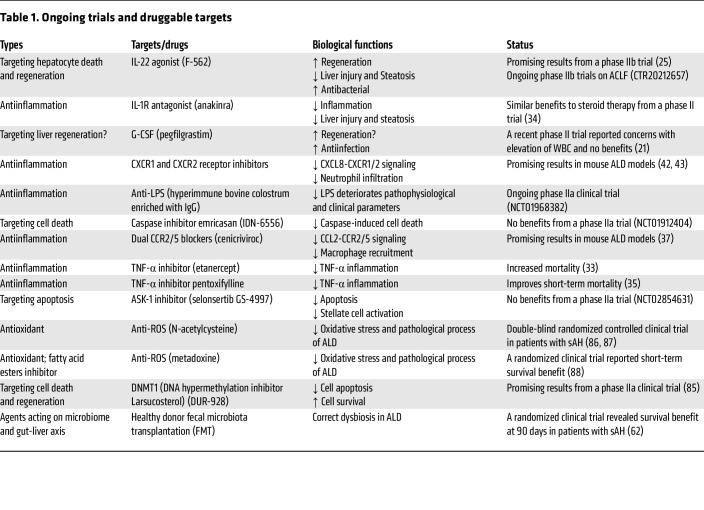
Ongoing trials and druggable targets

## Version 1 02/01/2024

Electronic publication
